# Unique Molecular Features in High-Risk Histology Endometrial Cancers

**DOI:** 10.3390/cancers11111665

**Published:** 2019-10-27

**Authors:** Pooja Pandita, Xiyin Wang, Devin E. Jones, Kaitlyn Collins, Shannon M. Hawkins

**Affiliations:** Department of Obstetrics and Gynecology, Indiana University School of Medicine, Indianapolis, IN 46202, USA; ppandita@iu.edu (P.P.); xw49@iu.edu (X.W.); devkenne@iu.edu (D.E.J.); kaicolli@indiana.edu (K.C.)

**Keywords:** uterine cancer, endometrial cancer, precision medicine, treatment, hormone therapy, stem cells, molecular features, high-risk histology

## Abstract

Endometrial cancer is the most common gynecologic malignancy in the United States and the sixth most common cancer in women worldwide. Fortunately, most women who develop endometrial cancer have low-grade early-stage endometrioid carcinomas, and simple hysterectomy is curative. Unfortunately, 15% of women with endometrial cancer will develop high-risk histologic tumors including uterine carcinosarcoma or high-grade endometrioid, clear cell, or serous carcinomas. These high-risk histologic tumors account for more than 50% of deaths from this disease. In this review, we will highlight the biologic differences between low- and high-risk carcinomas with a focus on the cell of origin, early precursor lesions including atrophic and proliferative endometrium, and the potential role of stem cells. We will discuss treatment, including standard of care therapy, hormonal therapy, and precision medicine-based or targeted molecular therapies. We will also discuss the impact and need for model systems. The molecular underpinnings behind this high death to incidence ratio are important to understand and improve outcomes.

## 1. Introduction

Endometrial cancer is the sixth most common malignancy in women, with over 380,000 new cases in women worldwide in 2018 [1]. In the United States, nearly 62,000 women will be diagnosed in 2019 [2]. Unfortunately, the incidence of endometrial cancer in the United States continues to increase [2]. Over 85% of women diagnosed with endometrial cancer will have low-grade endometrioid carcinomas with an all-stage 5-year survival of 83% [3]. High-risk histologic carcinomas such as uterine carcinosarcoma and high-grade endometrioid, serous, and clear cell carcinomas, are less prevalent, but are unfortunately significantly more deadly. High-risk carcinomas account for 15% of cases, but are responsible for up to 50% of deaths from endometrial cancer [4,5]. This review will highlight the biologic differences between low- and high-risk carcinomas, focusing on precursor lesions, stem cells, molecular targeted therapies, and model systems. Understanding the unique biology between the more easily cured low-grade tumors and the significantly deadly high-risk histologic carcinomas will impact outcomes.

## 2. Biologic Differences in Low- And High-Risk Histologic Endometrial Carcinomas

Historically, endometrial cancer has been categorized into two groups: type I tumors (low-grade) or type II tumors (high-grade). This system was based on clinical, endocrine, and epidemiological observations [6]. While this classification system has been used since the 1980s, increasing evidence suggests this classification is imperfect [7]. Molecular and pathologic factors are more frequently being used to classify endometrial cancer [8]. For simplicity, this review will focus on more common histopathology types, rather than the rarer. Rare tumors of the endometrium include neuroendocrine tumors and undifferentiated tumors. Similarly rare, dedifferentiated tumors consist of low-grade tumors adjacent to undifferentiated carcinoma. Case studies suggest these dedifferentiated tumors are more aggressive [9,10,11]. In this review, we will compare two general groups: low-risk histologic carcinomas and high-risk histologic carcinomas.

### 2.1. Clinical Features of Low- and High-Risk Histologic Carcinomas

Low-risk carcinomas are low-grade endometrioid carcinomas. High-risk histologic carcinomas include carcinomas with malignant stroma such as uterine carcinosarcomas and tumors with malignant epithelium including high-grade endometrioid, serous, and clear cell carcinomas. High-risk histologic carcinomas are clinically very different from low-grade endometrioid carcinomas. Table 1 highlights the general clinical features of low- and high-risk histologic endometrial carcinomas. First, high-risk histologic carcinomas are high-grade carcinomas [12]. High tumor grade is the most significant risk factor for subsequent death and disease recurrence [13,14]. Women with high-risk histologic carcinomas have poor outcomes (44% 5-year all-stage survival rate). Individuals with low-grade carcinomas have more favorable outcomes with an 83% 5-year survival rate for all stages [2,3]. Second, low-risk carcinomas are estrogen-dependent and associated with obesity. The excess estrogen produced by adipocytes stimulates endometrial epithelial cells, leading to the precursor lesion of endometrial hyperplasia [15]. Recent studies have highlighted the potential for counteracting the overabundance of estrogen-effects on the uterus with a levonorgestrel-containing intrauterine system to treat low-grade endometrial carcinoma in select women [16]. High-risk histologic carcinomas are not typically associated with excess estrogen or obesity. High-risk histologic carcinomas develop in a background of atrophic endometrium. Both low- and high-risk carcinomas have postmenopausal bleeding as the key presenting symptom, facilitating diagnosis at an early stage [17].

### 2.2. Health Disparities and High-Risk Histologic Carcinomas

Out of all cancers, endometrial carcinomas show the most considerable disparity due to race. The all-stage 5-year survival for non-Hispanic black women is 62%, and for non-Hispanic white women is 83% [2]. The incidence for both non-Hispanic white and non-Hispanic black women has remained stable when comparing linear trends from 1997 to 2014. However, black women have had a higher incidence of endometrial carcinoma since 2002 [18]. High-risk endometrial carcinoma has increased in both non-Hispanic white and black populations [18]. However, the death rate for non-Hispanic black women remains high. Death rates for non-Hispanic black women were 8.7 per 100,000 women for the years 2012–2016, while the death rate for non-Hispanic white women was 4.4 [19]. In addition to having two times the death rate, non-Hispanic black women are twice as likely to be diagnosed with high-risk disease [20]. Unfortunately, non-Hispanic black women are less likely to receive guideline-concordant clinical care for symptoms associated with endometrial carcinoma. Regrettably, women who do not receive guideline-concordant care are more likely to have high-risk carcinomas [21].

### 2.3. Pre-Cursor Lesions

Low-grade endometrioid carcinomas have a defined precursor lesion of endometrial hyperplasia. One of the major causes for the proliferation of the endometrial epithelial cells is high levels of estrogens, unopposed by progesterone. In women with significant obesity, high levels of estrogens can be found due to the conversion of androgens to estrogens through the aromatase in the adipose cells. For women with polycystic ovarian syndrome, unopposed estrogen is due to the reduced production of progesterone from anovulatory cycles, leading to hyperproliferation [22]. Endometrial epithelial cell hyperproliferation can lead to increased frequency of DNA damage, errors in DNA repair, and accumulation of mutations leading to further unchecked proliferation. Complex endometrial hyperplasia with atypia is the immediate precursor lesion for low-grade endometrioid carcinomas [15]. Reason suggests that uncontrolled proliferation may lead to additional errors in the DNA sequence that may allow low-grade disease to progress to high-risk histologic carcinomas, although this natural progression of the disease is not well studied.

High-risk histologic carcinomas develop within a background of atrophic endometrium. The mechanism by which the atrophic endometrium grows into these more rare carcinomas is less understood, and precursor lesions are not as clearly defined. Pathologic features of the precursor lesions were recently reviewed in [15], and some serous carcinomas have serous endometrial intraepithelial carcinoma as a precursor lesion. These lesions are confined to the surface of the endometrium without invasion and typically have mutations in p53. Unfortunately, there is no known precursor lesion for clear cell carcinoma, but some correlation would suggest that they may develop in endometrial polyps. Uterine carcinosarcomas have carcinomatous and sarcomatous components. A precursor lesion has not been identified in these components, but stem cells may play a role [15].

### 2.4. Stem Cells and Stem-Cell Markers

Stem cells are capable of self-renewal and the creation of mature cells through differentiation [23]. It is thought that each tissue type has a specialized stem cell. For example, hematopoietic stem cells (HSCs) are the precursors for blood and lymphoid cells. HSCs are likely to be the best-characterized stem cells, being studied within normal blood cell development and pathologies such as leukemias and lymphomas [24,25]. Stem cells play a significant role in normal endometrial physiology [26]. Through the menstrual cycle, the endometrium must undergo tissue repair, inflammation, and tissue regrowth in preparation for embryo implantation. In women with regular menstrual cycles, endometrial stem cells are required to supply new endometrial cells each month. Endometrial stem cells have the properties of stem cells, categorically, clonogenicity in colony-forming assays, long-term culturing capacity, ability to differentiate into different cell types in vitro, expression of stem cell markers, and in vivo reconstruction of tissue. Endometrial stem cells are thought to be located near the spiral arteries in the endometrium [26]. Endometrial stem cells have not been studied in atrophic endometrium. However, studies suggest that mesenchymal stem cell populations change during the perimenopausal period [27].

Intriguingly, studies have shown that bone marrow-derived stem cells can repopulate both mouse or human endometrium [26]. In women undergoing stem cell transplant, endometrial biopsies show donor HLA-type cells [28]. Recently, studies in mice have shown that bone marrow-derived stem cells can repopulate the mouse uterus and allow for fertility in previously subfertile mice [29]. In these experiments, Hoxa11-/- mice are infertile due to defects in decidualization, a form of endometrial stromal differentiation. Bone marrow transplant with Hoxa11+/+ bone marrow leads to stromal cells, which decidualized. Hoxa11+/- mice are subfertile. Bone marrow transplant with Hoxa11+/+ bone marrow rescues the subfertility [29]. Furthermore, bone marrow-derived stem cells can repopulate disease states such as endometriosis [30,31]. Ex vivo studies suggest several factors are important in endometrial regeneration. Recently, platelet-rich plasma has cellular effects on multiple endometrial cell types including stromal cells, mesenchymal stem cells, and bone marrow-derived mesenchymal stem cells. Platelet-rich plasma led to increased proliferation and migration of all cell types [32]. Repopulation of endometrial cells from bone marrow stem cells has implications for the development of precursor lesions and disease recurrence in endometrial carcinoma after hysterectomy, although this concept has not been studied experimentally. 

In recent decades, stem cells have come into focus in the study of cancer development, progression, and treatment via the cancer stem cell theory. The cancer stem cell theory proposes that within a tumor, there are small populations of cells capable of the self-renewing properties seen in normal stem cells [23,33]. However, unlike physiologically normal stem cells, cancer stems cells have a higher mutational burden, causing them to have increased dysfunction that may be related to their highly invasive properties. This dysfunctional signaling commonly occurs in growth signaling pathways frequently identified in cancers (i.e., *JAK/STAT, AKT, WNT, NFκB*), with particular activation in cells selected for “stemness” and may be responsible for the stem cell’s inadequate response to standard therapies [33,34]. Since cancer stem cells are less responsive to conventional therapies than the non-stem cell population within tumors, they are thought to survive following treatment, leading to cancer progression or recurrence. This resistance may be related to the mutational burden they possess or their slow cell division with increased DNA repair capability [33]. Due to their relationship to cancer development, progression, and treatment, further study of stem cells is necessary for many types of cancer including endometrial cancers, to better understand therapies to reduce recurrence of disease.

The expression of stem cell markers has been examined in endometrial carcinomas. Most studies have focused on endometrioid carcinomas or uterine carcinosarcomas. Early studies with endometrioid carcinomas showed that late-stage, high-grade disease maintained expression of epithelial stem cell markers while early-stage disease did not. Moreover, hierarchical clustering showed that tumors with retained expression of epithelial stem cell markers cluster most closely with late-stage disease [35]. Additionally, Musashi-1 is an RNA-binding protein that is thought to play a role in neural and epithelial progenitor cell proliferation and division. Examination of Musashi-1 staining shows that Musashi-1 staining is high in cells within the stem cell niche of the endometrium [36]. The stem/progenitor marker, CD133+, was determined to be a biomarker for poor prognosis tumors [37]. Isolation of cells from primary endometrioid carcinomas revealed that CD133+ cell populations had significant stem cell-like properties including small spheroid formation, chemotherapy resistance, clonogenicity, and the ability to form tumors in vivo [38]. Similarly, ALDH1-high cells isolated from primary endometrioid carcinomas exhibited similar stem-like properties [39]. Compared to work in other cancers such as ovarian cancer, there is relatively little known about stem cell characteristics in endometrial carcinomas.

Uterine carcinosarcoma histology is composed of malignant stroma and malignant epithelium. The cell of origin has been postulated as two different views: (1) an epithelial tumor collided with a stromal tumor with each tumor having a distinct cell of origin, or (2) both malignant stroma and epithelium being derived from a common stem cell precursor. The latter stem cell precursor hypothesis is known as the combination tumor theory. Most studies suggest that uterine carcinosarcomas arise via the combination tumor theory [40,41]. Studies have shown that CD133+ cells isolated from uterine carcinosarcomas have properties of cancer stem cells such as differentiation into both epithelial and stromal lineages, chemotherapy resistance, increased expression of stem cell markers including *BMI1*, and in vivo transplantable tumors [42]. Studies using the CS99 cell line, which is derived from uterine carcinosarcomas, showed that inhibition of *BMI1* (BMI1 proto-oncogene, polycomb ring finger) with the BMI1 inhibitor, PTC-028, led to increased apoptosis and decreased cell viability in vitro and delayed tumor growth in vivo [43]. These results suggest that targeting stem cells may be an effective treatment for uterine carcinosarcomas.

### 2.5. Unique Molecular Features

Histologic classification of high-grade endometrial carcinomas can be challenging as some carcinomas may exhibit characteristics of multiple histologic subtypes or a mixed phenotype. Detailed histologic classification schemes were recently summarized based on discussion at the Endometrial Cancer Workshop at the 2016 Society of Gynecologic Pathologists meeting [12]. In general, many carcinomas can be classified based on routine hematoxylin and eosin staining and microscopic examination. Molecular immunohistochemistry may be used when biopsy specimen does not allow an adequate evaluation of the whole tumor (i.e., endometrial biopsy) or to determine whether histology represents a focal area of a high-grade lesion or a pure high-grade carcinoma. The useful immunohistochemistry analyses are listed in Table 2.

In terms of molecular features, TCGA (The Cancer Genome Atlas) performed a large sample size (n = 373) integrated, genomic, and proteomic analysis of both endometrioid and serous endometrial carcinomas. This multi-platform analysis classified carcinomas into four categories: (1) polymerase-ε (POLE) ultramutated, with mutations in a subunit of DNA polymerase epsilon; (2) microsatellite instability hypermutated (MSI-H); (3) copy-number low, with a lower mutational load; and (4) copy-number high, with low mutation rates and extensive copy number variation [8]. Both high-grade endometrioid and serous carcinomas had significant downregulation of estrogen and progesterone receptor expression and mutations in *TP53*. Many low-grade endometrioid carcinomas contained mutations in *PTEN* and *ARID1A*. Additionally, high-grade serous endometrial carcinomas shared significant molecular features with high-grade serous ovarian cancer [8]. These results suggest that high-grade endometrioid and serous endometrial carcinomas are molecularly unique and should be treated differently in clinical trial analysis and design.

While the groundbreaking effort of TCGA detailed the genomic, transcriptomic, and proteomic landscape of low- and high-grade endometrioid and high-grade serous endometrial carcinomas [8], they did not evaluate high-grade clear cell endometrial carcinoma or uterine carcinosarcomas. Whole exome sequencing of uterine carcinosarcomas revealed that mutations in *TP53* were common (67%). Additionally, phosphoinositide 3-kinase pathway genes were frequently mutated including *PTEN*, *PIK3CA*, or *PIK3R1*. Loss of function mutations in chromatin remodeling genes including ARID1A and ARID1B were also common [57]. Whole exome sequencing of clear cell endometrial carcinomas similarly revealed high-frequency TP53 (39.7%), PIK3CA (23.8%), and ARID1A (15.9%) mutations. Clear cell endometrial carcinomas contained mutational features in common with both serous and endometrioid carcinomas [58]. Precision clinical trials that focus on unique molecular features of individual tumors are critical to improving outcomes [59]. Table 3 highlights the frequent genetic changes in each histology. Comprehensive analyses based on race and ethnicity are still missing.

## 3. Treatment

Currently, the standard of care treatment of endometrial carcinoma consists of surgery, radiation therapy, chemotherapy, or a combination of these [17,64]. Typically, treatment approaches are designed by a multidisciplinary tumor board, taking into consideration the National Comprehensive Cancer Network (NCCN) guidelines [64]. A recently published review highlights the treatment of advanced stage endometrial cancer [65]. Included in this are innovative molecular biology techniques that are focusing on clinically actionable changes in each woman’s carcinoma to direct therapy through clinical trials [66]. In molecularly targeted therapy, specific molecular targets that play a critical role in tumor progression are interrupted by medication, thus limiting harm to healthy cells. These are considered actionable targets because the identified molecular feature (i.e., the target) in a tumor has a specific therapy (i.e., the action) that works against tumors with that molecular feature. These genetic changes, known as actionable targets, are typically identified by next-generation sequencing of tumors or molecular immunohistochemistry (i.e., progesterone receptor, HER2) [67]. The Figure summarizes some of the more promising actionable targets and their specific therapies. Moreover, epigenetic modifying therapies hold some promise [68]. Summarized below is current information about primary treatment, hormonal treatment, current targeted therapy, and future targeted therapies.

### 3.1. Primary Surgical Therapy

The principle information needed for guiding the primary treatment of endometrial cancer is the surgical stage, grade, and histologic subtype [64]. Histologic type and grade of the tumor are first assigned using the endometrial biopsy or dilation and curettage specimen that provided the diagnosis of endometrial carcinoma. If the initial tissue assessment is concerning for high-risk carcinoma, then further testing with imaging and serum CA125 is recommended to assess extrauterine involvement before surgical staging. If instead, the initial tissue evaluation reveals low-grade endometrioid carcinoma, then women can go directly to surgical staging without further testing [64]. Surgical resection and staging are the mainstay of primary therapy in both high- and low-risk endometrial carcinomas. Surgery can serve as definitive management for low-risk, early-stage disease, and guides the decision for appropriate adjuvant treatment in later stages [64].

Comprehensive surgical staging in endometrial carcinoma includes total hysterectomy, bilateral salpingo-oophorectomy (TH/BSO), pelvic and para-aortic lymphadenectomy, collection of peritoneal cytology, and selective omental biopsy. If cervical involvement is suspected pre-operatively, then radical hysterectomy should be performed in place of total hysterectomy [64]. There is somewhat competing data in the literature about the value of full comprehensive staging, especially with regard to lymphadenectomy, in low-risk and early-stage carcinoma. A large, multi-country randomized trial showed that performing systematic pelvic lympadenectomy did not change overall survival or recurrence-free survival in the setting of low-risk cancer visually confined to the uterus on operative examination [69]. Based on this data and other supportive studies, the NCCN created criteria for when pelvic lymphadenectomy may not be necessary including tumor size less than 2 cm, less than 50% myometrial invasion, and well-differentiated histology [64]. In those cancers that do not meet these criteria, but still have low-risk histology and no operative evidence of extra-uterine disease, it may be appropriate to perform pelvic lymphadenectomy alone, with no para-aortic dissection [64].

Unlike with low-risk early-stage endometrial carcinoma, high-risk histology and late-stage disease have a much higher rate of metastasis and recurrence. Comprehensive surgical staging including pelvic and para-aortic lymphadenectomy should be performed in these cases, regardless of definitive evidence of extrauterine disease at the time of surgery [64,70]. Concerns for extra-uterine disease or nonresectable disease leads women toward neoadjuvant systemic therapy and/or primary pelvic radiation with reevaluation for subsequent response and surgical management [64].

### 3.2. Adjuvant Treatment

The indication for adjuvant therapy is based upon tumor histology, grade, and surgical stage. The majority of endometrial carcinomas are low-grade endometrioid carcinomas and do not require adjuvant therapy. NCCN guidelines recommend adjuvant therapy for high-risk histologic carcinomas [64]. There is no consensus on adjuvant therapy, but options include vaginal brachytherapy, external pelvic beam radiation therapy (EBRT), systemic chemotherapy, or a combination of these. Even with mixed evidence, the general principle stands that higher stage, higher grade, and higher risk histologic endometrial carcinomas are treated with more aggressive adjuvant therapy. For example, the GOG 99 study found that for low-grade endometrioid, stage Ib disease, adjuvant vaginal brachytherapy alone is recommended to reduce the risk of local recurrence [71].

There is no consensus on the ideal treatment for recurrent, metastatic, or high-risk disease. There are data to support a multimodal approach [17]. Options include surgical therapy, systemic chemotherapy, and radiotherapy [64]. Regimens may differ in type and number of chemotherapy medications, or the order of chemotherapy and radiation. As there are no clear cut guidelines for high-risk tumors, each case is often brought to a multidisciplinary tumor board meeting. The GOG-177 study was the first study to report a survival advantage to early proposed treatment regimens. Paclitaxel–doxorubicin–cisplatin was significantly superior to doxorubicin–cisplatin alone with regard to recurrence rates and overall survival, but the triplet regimen also had substantially higher neurotoxicity [72]. Since that time, carboplatin and paclitaxel have become the preferred regimen for systemic therapy. In the current literature, they have similar recurrence risk and survival outcomes to previously used multi-regimen therapies, but with much better tolerance [73,74]. The NCCN guidelines recommend carboplatin and paclitaxel as the preferred systemic chemotherapy regimen for the treatment of high-risk or recurrent endometrial cancer [64]. Further clinical trials are needed to understand the optimal adjuvant chemotherapy regimen, with both treatment and tolerance endpoints. Further investigation is also required for the evaluation of the ideal combination of chemotherapy and radiotherapy modalities for the primary treatment of high risk, advanced, and recurrent endometrial carcinoma.

### 3.3. Hormone Therapy

Hormone therapy with progestins and anti-estrogens are used for a variety of purposes in the treatment of endometrial cancer. Instead of hysterectomy, hormone therapy may be an option for select women who wish to preserve fertility. Studies have tested oral medroxyprogesterone acetate and levonorgestrel-containing intrauterine systems. The majority of women with low-grade endometrioid carcinomas, who are treated with a levonorgestrel-containing intrauterine system, had disease regression but not all [16]. Medroxyprogesterone acetate (MPA) is also used for fertility-sparing treatment. A study conducted in Japan found a complete response rate of 90.7% in women with early-stage low-grade endometrioid carcinoma, and 20% of women went on to have a successful pregnancy. However, 5-year recurrence-free survival was only 33% [75]. Thus, it is critical that women understand that fertility-sparing treatment is not a cure.

Progestin-based therapy has been widely used to treat advanced or recurrent endometrial cancer. In a phase II trial of high-dose megestrol acetate, 24% of patients showed a response. However, this response was not sustained, as only 31% of the responses lasted more than 18 months, and the median progression-free survival was only 2.5 months [76]. In a multicenter study based in Korea, medroxyprogesterone acetate treatment combined with a levonorgestrel-containing intrauterine system was evaluated in 35 women aged 27–40 with low-grade endometrioid endometrial cancer. After six months of treatment, 37.1% showed a complete regression, and 25.7% showed a partial response [77]. The expression of progesterone receptor was not examined in these tumors.

Hormonal therapy may also be a viable option for women with recurrent disease, women who require palliative care, or women who are poor surgical candidates. Assessment of steroid hormone receptor status (i.e., progesterone and estrogen receptor) can be clinically useful in these women. Decreased hormone efficacy in endometrial cancer has been proposed to be due to reduced PR expression due to selective hypermethylation [78,79]. In addition to progestin therapy, anti-estrogen regimens are an option [68]. Recently, a systemic review examined the response rate of women with advanced-stage or recurrent endometrial carcinomas to anti-estrogen treatments [80]. Anti-estrogens included tamoxifen, other selective estrogen receptor modulators, selective estrogen receptor downregulators, and aromatase inhibitors. All anti-estrogens as monotherapy had a statistically significant response rate. However, the effect of aromatase inhibitors was slight. Combination actionable target therapies such as mTOR inhibitors with an anti-estrogen, also had a statistically significant response. Not surprising, carcinomas which retained estrogen receptors had a higher response rate. While the response rates were statistically significant, the response rates were less than 50%, and there were limited effects on progression-free survival or overall survival [80]. Progestins and anti-estrogen therapies can be part of the systemic and locally-released treatments for endometrial carcinomas.

### 3.4. Targeted Therapy for HER2+ Tumors

An actionable target is an identified molecular feature in a cancer that has a specific treatment against that molecular feature. Amplification of ERBB2 (Erb-B2 receptor tyrosine kinase 2, also known as HER2) is an actionable target. ERBB2 is an epidermal growth factor receptor whose phosphorylation leads to the activation of proliferation and migration. Trastuzumab is a monoclonal antibody to ERBB2 that leads to antibody-dependent cytotoxicity. Overexpression of ERBB2 in early endometrial carcinomas is associated with disease recurrence [81]. Whole exome sequencing of clear cell endometrial carcinomas showed 11% of tumors harbored amplification in ERBB2 [82]. Serous endometrial carcinomas frequently have overexpression or amplification of ERBB2. Up to 53% of serous endometrial carcinomas have overexpression of ERBB2. Furthermore, some serous tumors had areas of both low and high HER2 expression [83,84]. As many endometrial carcinomas have this molecular feature that has a potential adjuvant therapy, some pathologists recommend routine immunohistochemistry staining for HER2 [83].

A case report of amplification of ERBB2 in uterine carcinosarcoma led to the treatment of a woman with trastuzumab and complete remission of recurrent disease [85]. Another study described the progression of disease on trastuzumab with paclitaxel in three women with HER2+ recurrent high-risk histology tumors including two serous and one endometrioid [86]. In vitro experiments in cells that overexpress ERBB2 showed that oncogenic mutations in PI3K resulted in resistance to afatinib, a tyrosine kinase inhibitor that inhibits ErbB signaling [87]. A randomized trial of systemic chemotherapy (carboplatin and paclitaxel) with trastuzumab in serous carcinomas increased median progression-free survival from 8.0 months to 12.6 months. The experimental treatment regimen was well tolerated and had no increase in toxicity [88]. Thus, ERBB2 remains an actionable target for some women.

### 3.5. Immune Therapy for MSI-H Carcinomas

Immune checkpoint inhibitors hold significant promise for women with endometrial carcinoma. Endometrial cancers are the most frequent cancers to have mismatch repair deficiency [89]. Furthermore, programmed cell death-1 (PD-1) and its ligand PD-L1 are highly overexpressed in primary and metastatic endometrial carcinomas [90]. This overexpression is associated with the microsatellite instability-high (MSI-H) status of tumors [91]. Without MSI testing, women with recurrent endometrial cancer who had failed multiple lines of testing were enrolled in a study to receive pembrolizumab, an immune checkpoint inhibitor, and lenvatinib, a VEGF receptor kinase inhibitor. The overall response rate was 48% [92]. A clinical trial examined the response of PD-1 blockade in patients with mismatch repair deficiency across different solid tumor types. Out of the 86 total participants, 15 participants had endometrial cancer, and three of the 15 (20%) showed a complete response to the pembrolizumab regimen. For the endometrial cancer patients, the objective response rate and disease control rate were 53% and 73%, respectively. The disease control rate was defined as the percentage of individuals who showed a complete response, partial response, or stable disease for 12 weeks or longer [89]. Thus, immune checkpoint inhibitors hold significant promise in women with endometrial carcinoma. However, there may be a need to determine patient selection characteristics based on the molecular features of the tumor.

### 3.6. Select Studies from Promising Molecular Targeted Therapies

NCCN guidelines recommend a genetic risk evaluation for all ovarian carcinomas, but those guidelines have not extended to endometrial carcinomas. A recent study examined the prevalence of detailed molecular characterization and the use of actionable treatments for gynecologic cancers including both ovarian and endometrial carcinomas. This dataset included 46 women with endometrial cancer. A majority of endometrial cancers (73%) had actionable targets identified. Low-risk tumors had more actionable mutations. After the discovery of actionable targets, treatment plans were changed in 56% of women with endometrial cancer. Most of the targeted therapies involved hormonal therapy (i.e., tamoxifen, letrozole, or medroxyprogesterone acetate) or mTOR inhibition (i.e., everolimus or temsirolimus) [67]. The frequency of actionable mutations was similar to the frequency of actionable mutations in ovarian cancer. Clinically, all ovarian cancers should have next-generation sequencing to determine actionable mutations. Endometrial cancer guidelines do not yet recommend this technology. However, this study highlights the fact that endometrial cancers have as many actionable targets identified that clinically change therapy and should be studied further [67].

As many endometrial cancers harbor mutations in PTEN, mammalian target of rapamycin (mTOR) inhibitors are a logical treatment option. The mTOR inhibitor temsirolimus has been studied in phase II trials in women with recurrent or metastatic endometrial cancer. In the group of 20 women who had not previously received chemotherapy, 14% showed a partial response, and 69% had stable disease. In the group of 25 women who had previously received chemotherapy, 4% showed a partial response, and 48% had stable disease. When compared to single-agent chemotherapeutic or hormonal agent regiments, temsirolimus had a lower proportion (17%) of patients that exhibited disease progression [93]. Unfortunately, studies of mTOR inhibitors (i.e., everolimus, temsirolimus, or ridaforolimus) have shown modest effects. These modest effects may be attributed to the crosstalk with the PI3K/AKT pathway and other pathways [65]. For example, KRAS mutations lead to resistance to everolimus [94]. Positively, an in vitro study suggests that inhibition of mTOR increases sensitivity to PARP inhibitors (i.e., olaparib, talazoparib, and BKM-120) [95].

Obesity and insulin resistance are known risk factors for endometrial cancer [17]. Insulin-like growth factor 1 (IGF-1) may have a role in the increased rates of endometrial cancer seen in diabetic patients. After binding of IGF-1 to its receptor IGF1-R, activation of the PI3K signaling pathway can occur, promoting cell growth, and inhibiting apoptosis. Inhibition of the IGF1-R pathway has been shown to have anticancer effects [96]. Metformin, conventional therapy for treating insulin resistance, likely acts similarly to mTOR inhibitors. Metformin activates AMP-activated protein kinase (AMPK), thereby regulating energy metabolism. AMPK is activated via phosphorylation by the kinase LKB1 and plays an active role in the regulation of the mTOR pathway [97]. Polycystic ovary syndrome (PCOS) is a multisystem disorder with reproductive, metabolic, and cardiovascular effects. Insulin resistance is a concern in individuals with PCOS, and reducing insulin levels has been associated with an improvement in menstrual dysfunction and reestablishment of ovulation cycles [98]. When given to women with PCOS, metformin resulted in restored ovulation and improved fertility [99]. When tested on endometrial cancer cell lines (ECC1 and Ishikawa), metformin treatment resulted in the inhibition of cell growth, induction of apoptosis, and decreased *TERT* (telomerase reverse transcriptase) expression, which is an oncogenic determinant [97]. Unfortunately, multiple ECC1 isolates have been genotyped as Ishikawa cells [100]. Others have shown the in vitro effects of metformin on Ishikawa cells including cell cycle arrest, apoptosis, sensitization to paclitaxel, and sensitization to progesterone [101,102,103]. A pooled meta-analysis of seven clinical studies showed that metformin reduced the risk of endometrial carcinoma recurrence (odds ratio (OR) = 0.5, 95% confidence interval (CI) 0.28–0.92, *p* < 0.05) and improved overall survival (hazard ratio (HR) = 0.61, 95% CI 0.48–0.77, *p* < 0.05). The overall survival was further enhanced in women with diabetes (HR = 0.47, 95% CI 0.33–0.67, *p* < 0.05) [104]. These are encouraging results for the use of metformin in women at high-risk for endometrial cancer as a preventative strategy and improving patient outcomes [97,99].

The poly-ADP ribosylation of DNA repair proteins is catalyzed by the enzyme poly(ADP-ribose) polymerase (PARP). PARP inhibitors were first designed to act against cells containing *BRCA1* or *BRCA2* mutations; however, clinical trials have indicated that these agents may be applicable in other cancers as cells that are deficient in *PTEN* have been shown to display sensitivity to PARP inhibitors [105]. Studies of endometrioid endometrial cancer showed that *PTEN*-deficient cell lines were not responsive to monotherapy of the PARP inhibitor, Olaparib. However, cell growth was inhibited in vitro when treated with the PARP inhibitior, Olaparib, combined with *PI3K* inhibition by BKM120. Importantly, this combination also showed inhibition of tumor growth in a genetically engineered mouse model of endometrial cancer (Ad^Cre^; *Pten^f/f^*; *Lkb1^f/f^)* [106].

*ARID1A* is frequently mutated in endometrial carcinomas (Table 3). Pre-clinical studies show that EZH2 inhibitors lead to the inhibition of ARID1A-mutant cancer cell lines via spheroid size and regression of tumors in vivo [107]. Similarly, studies in *ARID1A*-mutated ovarian cancer cell lines showed that the pan histone deacetylase inhibitor, suberoylanilide hydroxamine, also leads to decreased in vitro and in vivo tumor size [108]. Expanding beyond *ARID1A* to other members of the SWI/SNF complex subunit, Januario et al. showed that *SMARCA4*-mutant tumors were also sensitive to EZH2 inhibition, although no endometrial cancer cell lines were studied [109]. Furthermore, EZH2 inhibition in *ARID1A*-mutant cells may result in EZH2 inhibition that may be overcome by the addition of BCL2 inhibitors [110]. Understanding the complex synthetic lethality pathways and resistance pathways in other cancers may be useful toward treatments in similarly mutant endometrial cancers.

Mutations of epidermal growth factor receptor (EGFR) and its family are found in 43%-67% of endometrial carcinomas [111,112]. Erlotinib is a tyrosine kinase inhibitor for EGFR that likely leads to cell cycle arrest and angiogenesis inhibition [113]. A preclinical orthotopic mouse study showed the antitumor effects of erlotinib on both Ishikawa and HEC1A cells with a high expression of EGFR [114]. A phase II study of erlotinib for women with recurrent or advanced endometrial cancer showed an objective response rate of 12.5% and disease stabilization in nearly 50%. The response did not correlate with EGFR amplification or mutation. The authors speculate that erlotinib may be targeting EGFR, but affecting novel downstream pathways involved in PI3K signaling and progesterone resistance [113].

## 4. Model Systems

### 4.1. Mouse Models

Many genetically engineered mouse models of endometrial cancer have been created (Supplementary Table S1). However, none of the genetically engineered mouse models of endometrial cancer recapitulate the high-risk histologic carcinomas. Recently in Belgium, patient-derived xenograft (PDX) models were created from uterine carcinosarcomas. Out of seven samples, only two uterine carcinosarcomas engrafted. Importantly, tumors from these PDX models were molecularly characterized using molecular immunohistochemistry, copy number analysis, and RNA sequencing. Uterine carcinosarcomas maintained cytokeratin and vimentin staining in PDX when compared to the initial samples. Copy similarities ranged from 47.4–65.8%. A comparison of the transcriptomic profile of the original tumor sample to PDX showed that only one of the two samples clustered with its original tumor sample [115]. Similarly, PDX models of high-risk histologic tumors were created in China and compared to the original tumors using molecular immunohistochemistry and RNA-sequencing technology. Tumor samples from 18 women included ten high-grade endometrioid, six serous, one clear cell, and one carcinosarcoma. Interestingly, tumors were implanted into hormone depleted female mice. The clear cell carcinoma sample did not engraft, 2/6 serous carcinoma samples did not engraft, and one high-grade endometrioid carcinoma sample did not engraft. Only six PDX models had sufficient samples for immunophenotypic analysis. All PDX and original samples showed a similar expression of estrogen receptor, progesterone receptor, cytokeratin, and p53. Genomic analysis was only performed on two high-grade endometrioid samples. DNA mutation and transcriptomic changes correlated well between the original tumor and the PDX model [116]. Thus, additional genetically engineered mouse models of high-risk histologic carcinoma are needed. Furthermore, efforts to create PDX models of diverse racial and ethnic backgrounds will be necessary.

### 4.2. In Vitro Systems

Many human endometrial cancer cell lines have been created (Supplementary Table S2). Importantly, the CRISPR/Cas9 gene-editing tool has allowed for the development of engineered cancer cell models. The endonuclease Cas9 makes a double-strand break at the target site. The break can then be imperfectly repaired by the cellular mechanism non-homologous end joining, thereby introducing a mutation or gene knockout [117]. CRISPR technology was used to show that serum deprivation-response protein affects the expression of ALDH1 in HEC1B, HEC-108, HEC-116, and SNG-M cells. These studies essentially represent endometrioid carcinomas [118]. In serous carcinoma cell lines, CRISPR technology was used to introduce mutations in *FBXW7*, F-box, and WD repeat domain containing 7. Both the ARK1 and ARK2 cell lines with *FBXW7* mutations showed increased sensitivity to SRC inhibitors as well as dinaciclib, a CDK2 inhibitor [119]. The generation of such modified cell lines that genetically and phenotypically mimic cancer types allows for downstream functional studies and the potential to advance the field of targeted cancer therapeutics.

## 5. Future Systems

As an innovative technology, liquid biopsy has become an important diagnostic and prognostic test, moving in the direction of precision oncology [120,121]. This tool can detect circulating biomarkers such as circulating tumor DNA (ctDNA) and circulating free DNA (cfDNA) to gain information on the genetic basis of cancers, thereby diagnosing inaccessible tumors, predicting disease recurrence, assessing drug resistance, and providing direction for personalized treatment [120,122]. Additional advantages of the liquid biopsy include that it is minimally invasive, can provide early detection of cancer, and only requires up to 6–10 mL of blood [121]. Assessment of liquid biopsies has proven to be an accurate tool for cancers of the lung, prostate, and breast [123,124,125], but there have been few studies in comparison to gynecological cancers [126].

However, uterine lavage has been used to study proprotein convertases (PCs). PCs have a role in the cleavage of precursor proteins such as growth factors, hormones, and receptors, thereby activating them. PCs are associated with tumor development, though they are also found in normal tissue cells [127]. After assessing the expression of various PCs in the cell lines, only the upregulation of furin was consistently observed in all of the tested cell lines, providing evidence for its association with the development of endometrial carcinomas. Furthermore, endometrial cancer patients were found to have significantly increased total PC activity in uterine lavage when compared to the control patients [128,129]. This new, non-invasive technique of analyzing PC expression in uterine lavage fluid has been proposed as a new screening technique in postmenopausal women for early detection of endometrial cancer [127,128,129]. Additionally, PC inhibitors may play a future role in precision cancer therapy [127].

Additionally, there is ongoing research on using cervical fluid samples retrieved during routine Papanicolaou (Pap) as the basis of a new screening tool called PapSEEK [130]. The PapSEEK test is currently in development as an early endometrial and ovarian cancer diagnostic tool. This tool uses a cervical cytobrush to collect epithelial cells and the DNA that has been shed from the uterine lining. Next-generation sequencing is utilized to detect tumor DNA, potentially at a stage before cells are even available for histologic analysis [130]. Further studies are required to determine the optimal cytobrush. One limitation of these molecular assays is that similar mutations in benign and malignant endometrium may affect the test’s specificity. To complicate this even more, the endometrium is composed of both epithelial and stromal cells. Recent studies show that endometrial epithelium and stroma contain distinct mutation profiles, even from benign endometrium from the same woman [131]. Potential oncogenic mutations found included *PIK3CA*, *KRAS*, *PIK3R1*, and *FGFR2* in benign epithelial endometrium from women with uterine fibroids, and *ARID1A* mutations in endometrial stroma from women with endometriosis [131]. Finally, because the PapSEEK test is designed for both ovarian and endometrial cancer, clinical next steps have not been determined. As ovarian cancer screening algorithms have led to significant morbidity and mortality from next step diagnostic tests, these next steps are critical for the evaluation of a screening test [132]. Importantly, making screening and diagnosis less daunting may improve compliance with guideline-concordant care.

## 6. Conclusions

Despite medical developments, survival has not improved in women with endometrial cancer. While surgery alone or in combination with systemic or radiation therapy for confined diseases can be curative, individuals with metastatic or recurrent disease have reduced response rates to hormonal and chemotherapeutic agents. Further research on risk factors, genetics, and medical therapies is essential to counter the increasing death rates. In recent years, there have been widespread efforts toward incorporating molecular testing into the clinical evaluation and for these tests to serve as prognostic indicators. The identification of molecular aberrations, genetic mutations, and dysregulated pathways in endometrial carcinomas will be of great significance in the development of further immunotherapeutic and personalized therapeutic agents.

## Figures and Tables

**Table 1:** General clinical characteristics.

Characteristic	Low-Risk Histology (85%)	High-Risk Histology (15%)
**Characteristic histology**	Low-grade endometrioid	• Uterine carcinosarcoma • High-grade endometrioid • High-grade clear cell • High-grade serous
**5-year survival**	83%	44%
**Primary therapy**	• Removal of uterus and ovaries	• Removal of uterus and ovaries
	• Obesity may limit surgical options	• Aggressive tumor debulking
	• Fertility preservation with progesterone IUD	
**Adjuvant therapy**	Usually none	Chemotherapy+/-radiotherapy
**Timing of presentation**	• Young women (Lynch)	• Postmenopausal
	• Peri-menopausal	
	• Postmenopausal	
**Precursor lesion**	• Proliferative endometrium	• Atrophic endometrium
	• Endometrial hyperplasia	

**Table 2:** Markers.

Marker	Low-Grade Endometrioid	High-Grade Endometrioid	Clear Cell	Serous	Uterine Carcinosarcoma	References
**ARID1A-**	8%	33–46%	13–22%	0–9%	7%	[12,44,45,46]
**Estrogen receptor+**	84–96%	31–82%	9–21%	31–54%	8–36%	[12,44,47,48,49,50,51,52]
**HNF1β+**	39%	5%	67–83%	22%		[44,45]
**Napsin A+**	0%	0%	70–88%	8%	0%	[44,47,53]
**p16+**	1–7%	11–25%	45–80%	80–100%	60–78%	[12,47,48,49,51]
**p53+**	3–18%	18–69%	22–25%	44–94%	26–80%	[12,46,48,50,52,54,55]
**Progesterone receptor+**	83–100%	42–68%	11–45%	6–54%	0–42%	[12,44,47,48,49,51,52,55]
**PTEN-**	53%	28–75%	33%	6–100%	39%	[12,48,52]
**Vimentin+**	88–90%	77–92%	38–91%	27–83%	100%	[12,45,51]
**WT1+**	0–3%	0–27%	0–50%	33–63%	70%	[12,54,56]

**Table 3:** Common genetic changes.

Genetic Change	Low-Grade Endometrioid	High-Grade Endometrioid	Clear Cell	Serous	Uterine Carcinosarcoma	Reference
*ARID1A* mutation	46.7%	60%	14–22%	8–10.8%	12–23.8%	[15,58,60,61]
*CTNNB1* mutation	23.8%	20%	0%	1–2.7%	2–4.8%	[60,61,62]
*KRAS* mutation	15–24%	26.7%	0–13%	3–8.1%	9–29%	[15,60,61]
*PIK3CA* mutation	38–56%	56.7%	14–37%	17–43%	15–41%	[8,15,58,60]
*PTEN* mutation	67–80%	90%	0–25%	2.7–10%	18–47%	[15,60,61]
*TP53* mutation	10%	30%	31–50%	> 85%	64–91%	[8,15,60]
Microsatellite instability	30%	56%	0–19%	0%	3.5–21%	[15,58,62,63]

## Supplementary Materials

The following are available online at https://www.mdpi.com/2072-6694/11/11/1665/s1, Table S1: (A). Genes important in mouse models of endometrial cancer. (B) Genetically engineered endometrial cancer mouse models. Table S2. Endometrial cancer cell lines.
