# Supplementary material for: Unique Molecular Features in High-Risk Histology Endometrial Cancers
Source: Cancers (Basel). 2019 Oct 27;11(11):1665. doi: 10.3390/cancers11111665 (PMC6896116; doi:10.3390/cancers11111665)
Supplement: Supplementary file 1 [file cancers-11-01665-s001.zip › cancers-613465-supplementary.docx]

**Supplemental Materials**

Unique Molecular Features in High-Risk Histology Endometrial Cancers

Pooja Pandita, Xiyin Wang, Devin E. Jones, Kaitlyn E. Collins and Shannon M. Hawkins

**Table S1A.** Genes important in mouse models of endometrial cancer.

| Mouse Allele | Gene Name | Effect of Cre Recombination | References |
| --- | --- | --- | --- |
| *Alk5^f/f^* | Activin-like kinase 5 | Loss of ALK5 | [1] |
| *Ctnnb1^f(ex3)/+^* | beta-catenin | Dominant stabilized β-catenin | [2] |
| *Ctnnb1^f/f^* | beta-catenin | Loss of β-catenin | [2] |
| *Lkb1^L/L^* | Also name as Stk11 or Par4, serine/threonine kinase 11 | Inactivation of LKB1 | [3,4] |
| *Mig-6^f/f^* | Mitogen-inducible gene 6 | Loss of MIG-6 | [5,6] |
| *Pten^f/f^* | Phosphatase and tensin homolog | Loss of PTEN | [7] |
| ROSA^Pik3ca^ | phosphatidylinositol-4,5-bisphosphate 3-kinase catalytic subunit alpha | Activation of PI3K mutation | [8] |
| *Kras^G12D^* | KRAS Proto-Oncogene, GTPase | Expression oncogenic Kras | [9] |
| *P53^f/f^* | Tumor protein p53 | Loss of p53 | [7] |
| *Tgfbr1^f/f^* | Transforming growth factor beta receptor 1 | Loss of TGFBR1 | [10] |
| *Smad2^f/f^ Smad3^f/f^* | SMAD family member 2/3 | Loss of SMAD2/3 | [11] |
| *Spop^f/f^* | Speckle-type poz protein | Loss of SPOP | [12] |

**Table S1B.** Genetically engineered endometrial cancer mouse models.

| **Genotype** | **Phenotype** | **Penetrance** | **References** |
| --- | --- | --- | --- |
| *Alk5^f/f^Pgr^cre+^* | Endometrial adenocarcinoma with metastasis to the lungs | 5/14 of females (mated to fertile males) with endometrial adenocarcinoma 0/3 of virgin females | [1] |
| *Cables^−/−^* | Endometrial hyperplasia | 20/20 with endometrial hyperplasia from 3 to 6 months | [13] |
| *Ctnnb1^f(ex3)/+^Pgr^cre+^* | Endometrial hyperplasia | Endometrial hyperplasia at 6 weeks | [2] |
| *Ctnnb1^f/f^Pgr^cre+^* | Squamous cell metaplasia | Squamous cell metaplasia at 6 weeks | [2] |
| *Dab2^+/−^* | Endometrial hyperplasia | 48/133 with endometrial hyperplasia from 6 to 12 months | [14] |
| *Lkb^−/+^* | Endometrial adenocarcinoma | 8/15 with endometrial adenocarcinoma by 55 weeks | [3] |
| *Lkb1^L/L^ Sprr2f^cre+^* | Endometrial adenocarcinoma | Endometrial adenocarcinoma after 12 weeks | [4] |
| *Lkb1^L/L^Ade^cre^* | Endometrial adenocarcinoma | 11/17 with endometrial adenocarcinoma at 9 months | [3] |
| *Mig-6^f/f^Sprr2f^cre+^* | Endometrial hyperplasia | Endometrial hyperplasia after 10 weeks | [5] |
| *Mig-6^f/f^Wnt7a^cre+^* | Endometrial hyperplasia | 5/5 with endometrial hyperplasia by 5 months | [6] |
| *Par4^−/−^* | Endometrial carcinoma | 5/14 with endometrial carcinoma from 18 to 24 months | [15] |
| *Pten^+/−^* | Complex atypical hyperplasia and endometrial carcinoma | 8/8 with complex atypical hyperplasia and 2/8 with endometrial carcinoma at 40 weeks; 65/65 with endometrial hyperplasia and 14/65 with endometrial carcinoma from 6 months | [16,17] |
| *Pten^+/−^Mlh1^−/−^* | Complex atypical hyperplasia and endometrial carcinoma | 5/5 with complex atypical hyperplasia and 2/5 with endometrial carcinoma at 14-18 weeks | [16] |
| *Pten^f/f^/Ltf^Cre/+^* | Endometrial adenocarcinoma | 5/7 with endometrial adenocarcinoma after 6 months | [18] |
| *Pten^f/f^Amhr2^cre/+^* | No tumor | No tumor | [19] |
| *Pten^f/f^Ctnnb1^f/f^ROSA^Pik3ca^Ad^cre^* | Endometrioid endometrial adenocarcinoma | 4/4 of ovariectomized mice with endometrioid endometrial adenocarcinoma | [8] |
| *Pten^f/f^Kras^G12D^Pgr^cre+^* | Endometrioid endometrial adenocarcinoma | Endometrioid endometrial adenocarcinoma by 4 weeks | [9] |
| *Pten^f/f^Mig-6^f/f^Pgr^cre+^* | Endometrial adenocarcinoma | 8/8 with endometrial adenocarcinoma at 4 weeks | [20] |
| *Pten^f/f^P53^f/f^Pgr^cre+^* | Endometrial adenocarcinoma | 6/6 with endometrial adenocarcinoma by 2 months | [7] |
| *Pten^f/f^Pgr^cre+^* | Endometrial adenocarcinoma | 7/9 with endometrial adenocarcinoma by 3 months | [7] |
| *Pten^f/f^Tgfbr1^f/f^Pgr^cre+^* | Endometrial adenocarcinoma with metastasis to the lungs | 16/16 with endometrial adenocarcinoma by after 7 weeks | [10] |
| *Pten^L/L^Lkb1^L/L^Ad^cre^* | Endometrioid endometrial adenocarcinoma | Endometrioid endometrial adenocarcinoma | [21] |
| *Smad2^f/f^Smad3^f/f^Pgr^cre+^* | Endometrial adenocarcinoma with metastasis to the lungs | 9/9 with endometrial adenocarcinoma by 5 months | [11] |
| *Spop^f/f^Pgr^cre+^* | No tumor | 5/5 with dilated cystic uterine glands by 10 months | [12] |

**Table 2.** Endometrial cancer cell lines.

| **Cell Line** | **Original Derivation** | **Common or major mutations** | **Steroid hormone receptor expression** | **Ability to form tumors in mouse** | **References** |
| --- | --- | --- | --- | --- | --- |
| AN3 CA | Endometrioid adenocarcinoma (lymph node metastasis) (undifferentiated) (grade 3) | MAPK3, PIK3R1, PTEN, P53, ARID1A, FGFR2 | ER- | Yes | [22–26] |
| ECC-1 | Endometrioid adenocarcinoma (well-differentiated) *Contaminated. Shown to be a Ishikawa derivative | PTEN, TP53 | ER+, PR+, AR+ | Yes | [25,27,28] |
| EFE184 | Endometrioid adenocarcinoma | TP53 | NA | NA | [24,29] |
| EN | Endometrioid adenocarcinoma | PIK3CA, PTEN, FGFR2 | NA | NA | [24,26,29] |
| EN1 | Endometrioid adenocarcinoma | PIK3CA, PTEN | NA | NA | [26,29] |
| ESS-1 | Uterine carcinosarcoma | PIK3CA, PTEN, TP53, | NA | NA | [23,30] |
| HEC-108 | Endometrioid adenocarcinoma (grade 3) (undifferentiated) | PTEN | NA | Yes | [31,32] |
| HEC-116 | Endometrioid adenocarcinoma (well-differentiated) (grade 2) | NA | NA | Yes | [33] |
| HEC-151 | Endometrioid adenocarcinoma (grade 2) | PTEN, PIK3CA, NRAS | NA | NA | [32,33] |
| HEC-155 | Papillary serous adenocarcinoma *Contaminated | NA | NA | NA | [33–35] |
| HEC-161 | Endometrioid adenocarcinoma | PIK3CA, TP53 | NA | NA | [32,36] |
| HEC-180 | Papillary serous adenocarcinoma *Contaminated | NA | NA | NA | [34,35] |
| HEC-1-A | Endometrioid adenocarcinoma (Grade 2) (moderately well-differentiated) | PIK3CA, KRAS, TP53 | ER+, PR+ | Yes | [24,37–39] |
| HEC-1-B | Endometrioid adenocarcinoma (Grade 2) (moderately well-differentiated) | KRAS, TP53, PIK3CA | ER+, PR- | Yes | [24,28,37,39] |
| HEC-251 | Endometrioid adenocarcinoma (moderately well-differentiated) | PTEN, PIK3CA | NA | Yes | [32,40] |
| HEC-265 | Endometrioid adenocarcinoma (well-differentiated) | PTEN | PR+ | Yes | [32,40] |
| HEC-50 | Endometrioid adenocarcinoma (grade 3) | NA | ER- | NA | [25,33] |
| HEC-50B | Endometrioid adenocarcinoma | KRAS | NA | NA | [32] |
| HEC-50co | Papillary serous adenocarcinoma (undifferentiated) | KRAS, TP53 | ER-, PR- | Yes, | [41–45] |
| HEC-59 | Endometrioid adenocarcinoma (grade 2) | PTEN, PIK3CA | NA | Yes | [24,32,33] |
| HEC-6 | Endometrioid adenocarcinoma | PTEN, PIK3CA | NA | NA | [32,33] |
| HHUA | Endometrioid adenocarcinoma (grade 1) (well-differentiated) | KRAS, PTEN, PIK3CA | ER+, PR+ | Yes | [31,32,46] |
| HOOUA | Endometrioid adenocarcinoma (undifferentiated) | NA | NA | Yes | [31] |
| Ishikawa | Endometrioid adenocarcinoma (Primary) (well-differentiated) (grade 1) | PTEN, RB1, TP53, BRCA1, ARID1A | ER+, PR+, AR+ | Yes | [23,24,28,47] |
| JHUEM-1 | Endometrioid adenocarcinoma (grade 2) | PTEN, PIK3CA, ARID1A | NA | NA | [23] |
| JHUEM-2 | Endometrioid adenocarcinoma | PTEN, PIK3CA, ARID1A | NA | NA | [23] |
| JHUEM-3 | Endometrioid adenocarcinoma | NA | NA | NA | [23] |
| JHUEM-7 | Endometrioid adenocarcinoma | PTEN, PIK3CA, | NA | NA | [23] |
| KLE | Endometrioid adenocarcinoma (lymph node metastasis) (undifferentiated) | TP53 | ER- | Yes | [25,44,48] |
| MFE-280 | Endometrioid partly papillary adenocarcinoma (Recurrent) (grade 3) | PIK3CA, FGFR2 | ER+, PR+ | No | [26,44,49] |
| MFE-294 | Endometrioid partly papillary adenocarcinoma (grade 2) | NA | PR+ | NA | [29] |
| MFE-296 | Endometrioid adenocarcinoma (moderately well-differentiated) (primary) (grade 2) | PIK3CA, PTEN, FGFR2, TP53 | ER+, PR+, AR+ | Yes | [26,29,49,50] |
| MFE319 | Adenosquamous carcinoma (grade 1-2) | PTEN, FGFR2 | NA | NA | [29,49] |
| Nou-1 | Endometrioid adenocarcinoma | No PTEN, KRAS, PIK3CA | NA | NA | [24,32] |
| RL95-2 | Adenosquamous carcinoma (Grade 2) (moderately well-differentiated) | PTEN, TP53 | ER+ | Yes | [24,44,51,52] |
| SK-UT-2 | Adenocarcinoma (undifferentiated) | NA | ER+, PR+ | Yes | [53] |
| SNG-II | Endometrioid adenocarcinoma (grade 1) (well-differentiated) | PTEN, KRAS | ER- | Yes | [26,29,31,54] |
| SNG-M | Endometrioid adenocarcinoma (lymph node metastasis) (grade 2) (moderately well-differentiated) | B2M, KRAS, PIK3CA, PTEN | ER-, PR- | Yes | [26,29,31,46,55] |
| SNU-1077 | Uterine carcinosarcoma | TP53 | NA | NA | [23,56] |
| SNU-685 | Uterine carcinosarcoma | TP53 | NA | NA | [23,56] |
| SPAC-1-L | Serous adenocarcinoma | PTEN | NA | Yes | [29,57] |
| SPAC-1-S | Serous adenocarcinoma | NA | NA | Yes | [29,57] |
| SPEC-2 | Serous adenocarcinoma | TP53, No PTEN | NA | NA | [44,58,59] |
| TEN | Clear cell carcinoma | PIK3CA, KRAS, PTEN, TP53, RB1 | NA | NA | [23,60] |
| USPC1 | Serous adenocarcinoma | PIK3CA | NA | NA | [29,61] |
| USPC2 | Serous adenocarcinoma | NA | NA | NA | [29,61] |
| USPC-ARK-1, USPC-ARK-9-10, USPC-ARK-14, USPC-ARK-20 | Serous adenocarcinoma | PIK3CA | NA | NA | [62,63] |
| USPC-ARK-2-8, USPC-ARK-11-13, USPC-ARK-15-19, USPC-ARK-21-22 | Serous adenocarcinoma | PIK3CA not detected mutation | NA | NA | [62,63] |

References

1. Monsivais, D.; Peng, J.; Kang, Y.; Matzuk, M.M. Activin-like kinase 5 (alk5) inactivation in the mouse uterus results in metastatic endometrial carcinoma. *Proc. Natl. Acad. Sci. USA* **2019**, *116*, 3883–3892.

2. Jeong, J.W.; Lee, H.S.; Franco, H.L.; Broaddus, R.R.; Taketo, M.M.; Tsai, S.Y.; Lydon, J.P.; DeMayo, F.J. Beta-catenin mediates glandular formation and dysregulation of beta-catenin induces hyperplasia formation in the murine uterus. *Oncogene* **2009**, *28*, 31–40.

3. Contreras, C.M.; Gurumurthy, S.; Haynie, J.M.; Shirley, L.J.; Akbay, E.A.; Wingo, S.N.; Schorge, J.O.; Broaddus, R.R.; Wong, K.K.; Bardeesy, N.; et al. Loss of lkb1 provokes highly invasive endometrial adenocarcinomas. *Cancer Res.* **2008**, *68*, 759–766.

4. Contreras, C.M.; Akbay, E.A.; Gallardo, T.D.; Haynie, J.M.; Sharma, S.; Tagao, O.; Bardeesy, N.; Takahashi, M.; Settleman, J.; Wong, K.K.; et al*.* Lkb1 inactivation is sufficient to drive endometrial cancers that are aggressive yet highly responsive to mtor inhibitor monotherapy. *Dis. Model. Mech.* **2010**, *3*, 181–193.

5. Yoo, J.Y.; Kang, H.B.; Broaddus, R.R.; Risinger, J.I.; Choi, K.C.; Kim, T.H. Mig-6 suppresses endometrial epithelial cell proliferation by inhibiting phospho-akt. *BMC Cancer* **2018**, *18*, 605.

6. Kim, T.H.; Lee, D.K.; Cho, S.N.; Orvis, G.D.; Behringer, R.R.; Lydon, J.P.; Ku, B.J.; McCampbell, A.S.; Broaddus, R.R.; Jeong, J.W. Critical tumor suppressor function mediated by epithelial mig-6 in endometrial cancer. *Cancer Res.* **2013**, *73*, 5090–5099.

7. Daikoku, T.; Hirota, Y.; Tranguch, S.; Joshi, A.R.; DeMayo, F.J.; Lydon, J.P.; Ellenson, L.H.; Dey, S.K. Conditional loss of uterine pten unfailingly and rapidly induces endometrial cancer in mice. *Cancer Res.* **2008**, *68*, 5619–5627.

8. Terakawa, J.; Serna, V.A.; Taketo, M.M.; Daikoku, T.; Suarez, A.A.; Kurita, T. Ovarian insufficiency and ctnnb1 mutations drive malignant transformation of endometrial hyperplasia with altered pten/pi3k activities. *Proc. Natl. Acad. Sci. USA* **2019**.

9. Kim, T.H.; Wang, J.; Lee, K.Y.; Franco, H.L.; Broaddus, R.R.; Lydon, J.P.; Jeong, J.W.; Demayo, F.J. The synergistic effect of conditional pten loss and oncogenic k-ras mutation on endometrial cancer development occurs via decreased progesterone receptor action. *J. Oncol.* **2010**, *2010*, 139087.

10. Gao, Y.; Lin, P.; Lydon, J.P.; Li, Q. Conditional abrogation of transforming growth factor-beta receptor 1 in pten-inactivated endometrium promotes endometrial cancer progression in mice. *J. Pathol.* **2017**, *243*, 89–99.

11. Kriseman, M.; Monsivais, D.; Agno, J.; Masand, R.P.; Creighton, C.J.; Matzuk, M.M. Uterine double-conditional inactivation of SMAD2 and SMAD3 in mice causes endometrial dysregulation, infertility, and uterine cancer. *Proc. Natl. Acad. Sci. USA* **2019**, *116*, 3873–3882.

12. Hai, L.; Szwarc, M.M.; He, B.; Lonard, D.M.; Kommagani, R.; DeMayo, F.J.; Lydon, J.P. Uterine function in the mouse requires speckle-type poz protein. *Biol. Reprod.* **2018**, *98*, 856–869.

13. Zukerberg, L.R.; DeBernardo, R.L.; Kirley, S.D.; D'Apuzzo, M.; Lynch, M.P.; Littell, R.D.; Duska, L.R.; Boring, L.; Rueda, B.R. Loss of cables, a cyclin-dependent kinase regulatory protein, is associated with the development of endometrial hyperplasia and endometrial cancer. *Cancer Res.* **2004**, *64*, 202–208.

14. Yang, D.H.; Fazili, Z.; Smith, E.R.; Cai, K.Q.; Klein-Szanto, A.; Cohen, C.; Horowitz, I.R.; Xu, X.X. Disabled-2 heterozygous mice are predisposed to endometrial and ovarian tumorigenesis and exhibit sex-biased embryonic lethality in a p53-null background. *Am. J. Pathol.* **2006**, *169*, 258–267.

15. Garcia-Cao, I.; Duran, A.; Collado, M.; Carrascosa, M.J.; Martin-Caballero, J.; Flores, J.M.; Diaz-Meco, M.T.; Moscat, J.; Serrano, M. Tumour-suppression activity of the proapoptotic regulator par4. *Embo. Rep.* **2005**, *6*, 577–583.

16. Wang, H.; Douglas, W.; Lia, M.; Edelmann, W.; Kucherlapati, R.; Podsypanina, K.; Parsons, R.; Ellenson, L.H. DNA mismatch repair deficiency accelerates endometrial tumorigenesis in pten heterozygous mice. *Am. J. Pathol.* **2002**, *160*, 1481–1486.

17. Stambolic, V.; Tsao, M.S.; Macpherson, D.; Suzuki, A.; Chapman, W.B.; Mak, T.W. High incidence of breast and endometrial neoplasia resembling human cowden syndrome in pten+/- mice. *Cancer Res.* **2000**, *60*, 3605–3611.

18. Liang, X.; Daikoku, T.; Terakawa, J.; Ogawa, Y.; Joshi, A.R.; Ellenson, L.H.; Sun, X.; Dey, S.K. The uterine epithelial loss of pten is inefficient to induce endometrial cancer with intact stromal pten. *Plos Genet.* **2018**, *14*, e1007630.

19. Daikoku, T.; Jackson, L.; Besnard, V.; Whitsett, J.; Ellenson, L.H.; Dey, S.K. Cell-specific conditional deletion of pten in the uterus results in differential phenotypes. *Gynecol. Oncol.* **2011**, *122*, 424–429.

20. Kim, T.H.; Franco, H.L.; Jung, S.Y.; Qin, J.; Broaddus, R.R.; Lydon, J.P.; Jeong, J.W. The synergistic effect of mig-6 and pten ablation on endometrial cancer development and progression. *Oncogene* **2010**, *29*, 3770–3780.

21. Cheng, H.; Liu, P.; Zhang, F.; Xu, E.; Symonds, L.; Ohlson, C.E.; Bronson, R.T.; Maira, S.M.; Di Tomaso, E.; Li, J.; et al. A genetic mouse model of invasive endometrial cancer driven by concurrent loss of pten and lkb1 is highly responsive to mtor inhibition. *Cancer Res.* **2014**, *74*, 15–23.

22. Dawe, C.J.; Banfield, W.G.; Morgan, W.D.; Slatick, M.S.; Curth, H.O. Growth in continuous culture, and in hamsters, of cells from a neoplasm associated with acanthosis nigricans. *J. Natl. Cancer Inst.* **1964**, *33*, 441–456.

23. Barretina, J.; Caponigro, G.; Stransky, N.; Venkatesan, K.; Margolin, A.A.; Kim, S.; Wilson, C.J.; Lehar, J.; Kryukov, G.V.; Sonkin, D.; et al. The cancer cell line encyclopedia enables predictive modelling of anticancer drug sensitivity. *Nature* **2012**, *483*, 603–607.

24. Dedes, K.J.; Wetterskog, D.; Mendes-Pereira, A.M.; Natrajan, R.; Lambros, M.B.; Geyer, F.C.; Vatcheva, R.; Savage, K.; Mackay, A.; Lord, C.J.; et al. Pten deficiency in endometrioid endometrial adenocarcinomas predicts sensitivity to parp inhibitors. *Sci. Transl. Med.* **2010**, *2*, 53ra75.

25. Korch, C.; Spillman, M.A.; Jackson, T.A.; Jacobsen, B.M.; Murphy, S.K.; Lessey, B.A.; Jordan, V.C.; Bradford, A.P. DNA profiling analysis of endometrial and ovarian cell lines reveals misidentification, redundancy and contamination. *Gynecol. Oncol.* **2012**, *127*, 241–248.

26. Konecny, G.E.; Kolarova, T.; O'Brien, N.A.; Winterhoff, B.; Yang, G.; Qi, J.; Qi, Z.; Venkatesan, N.; Ayala, R.; Luo, T.; et al. Activity of the fibroblast growth factor receptor inhibitors dovitinib (tki258) and nvp-bgj398 in human endometrial cancer cells. *Mol. Cancer* **2013**, *12*, 632–642.

27. Mo, B.; Vendrov, A.E.; Palomino, W.A.; DuPont, B.R.; Apparao, K.B.; Lessey, B.A. Ecc-1 cells: A well-differentiated steroid-responsive endometrial cell line with characteristics of luminal epithelium. *Biol. Reprod* **2006**, *75*, 387–394.

28. Wang, Y.; Yang, D.; Cogdell, D.; Hu, L.; Xue, F.; Broaddus, R.; Zhang, W. Genomic characterization of gene copy-number aberrations in endometrial carcinoma cell lines derived from endometrioid-type endometrial adenocarcinoma. *Technol. Cancer Res. Treat.* **2010**, *9*, 179–189.

29. Konecny, G.E.; Venkatesan, N.; Yang, G.; Dering, J.; Ginther, C.; Finn, R.; Rahmeh, M.; Fejzo, M.S.; Toft, D.; Jiang, S.W.; et al*.* Activity of lapatinib a novel her2 and egfr dual kinase inhibitor in human endometrial cancer cells. *Br. J. Cancer* **2008**, *98*, 1076–1084.

30. Gunawan, B.; Braun, S.; Cortes, M.J.; Bergmann, F.; Karl, C.; Fuzesi, L. Characterization of a newly established endometrial stromal sarcoma cell line. *Int. J. Cancer* **1998**, *77*, 424–428.

31. Mikami, M.; Harasawa, M.; Sugiyama, T.; Nishijima, Y.; Goto, Y.; Hirasawa, T.; Muramatsu, T.; Iwamori, M. Induction of the differentiation of cultured endometrial carcinoma cells by type i collagen: Relevance of sulfolipids. *Oncol. Lett.* **2010**, *1*, 113–117.

32. Weigelt, B.; Warne, P.H.; Lambros, M.B.; Reis-Filho, J.S.; Downward, J. Pi3k pathway dependencies in endometrioid endometrial cancer cell lines. *Clin. Cancer Res.* **2013**, *19*, 3533–3544.

33. Kuramoto, H.; Nishida, M.; Morisawa, T.; Hamano, M.; Hata, H.; Kato, Y.; Ohno, E.; Iida, T. Establishment and characterization of human endometrial cancer cell lines. *Ann. NY Acad. Sci.* **1991**, *622*, 402–421.

34. Iida, T.; Hamano, M.; Yoshida, N.; Yonamine, K.; Hayashi, K.; Kiguchi, K.; Ishizuka, B.; Nishimura, Y.; Arai, T.; Kawaguchi, M.; et al. Establishment and characterization of two cell lines (hec-155, hec-180) derived from uterine papillary serous adenocarcinoma. *Eur. J. Gynaecol. Oncol.* **2004**, *25*, 423–427.

35. Capes-Davis, A.; Theodosopoulos, G.; Atkin, I.; Drexler, H.G.; Kohara, A.; MacLeod, R.A.; Masters, J.R.; Nakamura, Y.; Reid, Y.A.; Reddel, R.R.; et al. Check your cultures! A list of cross-contaminated or misidentified cell lines. *Int. J. Cancer.* **2010**, *127*, 1–8.

36. Kamata, Y.; Watanabe, J.; Hata, H.; Hamano, M.; Kuramoto, H. Quantitative study on the correlation between p53 gene mutation and its expression in endometrial carcinoma cell lines. *Eur. J. Gynaecol. Oncol.* **2004**, *25*, 55–60.

37. Kurarmoto, H.; Hamano, M.; Imai, M. Hec-1 cells. *Hum. Cell* **2002**, *15*, 81–95.

38. Castro-Rivera, E.; Safe, S. Estrogen- and antiestrogen-responsiveness of hec1a endometrial adenocarcinoma cells in culture. *J. Steroid. Biochem. Mo.l Biol.* **1998**, *64*, 287–295.

39. Horne, A.W.; Lalani, E.N.; Margara, R.A.; White, J.O. The effects of sex steroid hormones and interleukin-1-beta on muc1 expression in endometrial epithelial cell lines. *Reproduction* **2006**, *131*, 733–742.

40. Fujisawa, T.; Hamano, M.; Hata, H.; Kamata, Y.; Watanabe, J.; Sekimoto, R.; Kuramoto, H. Establishment and characterization of two different types of new human endometrial adenocarcinoma cell lines (hec-251 and hec-265). *Eur. J. Gynaecol. Oncol.* **2004**, *25*, 299–304.

41. Kumar, N.S.; Richer, J.; Owen, G.; Litman, E.; Horwitz, K.B.; Leslie, K.K. Selective down-regulation of progesterone receptor isoform b in poorly differentiated human endometrial cancer cells: Implications for unopposed estrogen action. *Cancer Res.* **1998**, *58*, 1860–1865.

42. Albitar, L.; Pickett, G.; Morgan, M.; Davies, S.; Leslie, K.K. Models representing type I and type II human endometrial cancers: Ishikawa h and hec50co cells. *Gynecol. Oncol.* **2007**, *106*, 52–64.

43. Dai, D.; Albitar, L.; Nguyen, T.; Laidler, L.L.; Singh, M.; Leslie, K.K. A therapeutic model for advanced endometrial cancer: Systemic progestin in combination with local adenoviral-mediated progesterone receptor expression. *Mol. Cancer* **2005**, *4*, 169–175.

44. Van Nyen, T.; Moiola, C.P.; Colas, E.; Annibali, D.; Amant, F. Modeling endometrial cancer: Past, present, and future. *Int. J. Mol. Sci.* **2018**, *19*, 2348.

45. Albitar, L.; Pickett, G.; Morgan, M.; Wilken, J.A.; Maihle, N.J.; Leslie, K.K. Egfr isoforms and gene regulation in human endometrial cancer cells. *Mol. Cancer* **2010**, *9*, 166.

46. Shimizu, H.; Inoue, M.; Tanizawa, O. Adoptive cellular immunotherapy to the endometrial carcinoma cell line xenografts in nude mice. *Gynecol Oncol* **1989**, *34*, 195–199.

47. Nishida, M.; Kasahara, K.; Kaneko, M.; Iwasaki, H.; Hayashi, K. Establishment of a new human endometrial adenocarcinoma cell line, ishikawa cells, containing estrogen and progesterone receptors. *Nihon Sanka Fujinka Gakkai Zasshi* **1985**, *37*, 1103–1111.

48. Richardson, G.S.; Dickersin, G.R.; Atkins, L.; MacLaughlin, D.T.; Raam, S.; Merk, L.P.; Bradley, F.M. Kle: A cell line with defective estrogen receptor derived from undifferentiated endometrial cancer. *Gynecol. Oncol.* **1984**, *17*, 213–230.

49. Hackenberg, R.; Hawighorst, T.; Hild, F.; Schulz, K.D. Establishment of new epithelial carcinoma cell lines by blocking monolayer formation. *J. Cancer Res. Clin. Oncol.* **1997**, *123*, 669–673.

50. Eritja, N.; Domingo, M.; Dosil, M.A.; Mirantes, C.; Santacana, M.; Valls, J.; Llombart-Cussac, A.; Matias-Guiu, X.; Dolcet, X. Combinatorial therapy using dovitinib and ici182.780 (fulvestrant) blocks tumoral activity of endometrial cancer cells. *Mol. Cancer* **2014**, *13*, 776–787.

51. Way, D.L.; Grosso, D.S.; Davis, J.R.; Surwit, E.A.; Christian, C.D. Characterization of a new human endometrial carcinoma (rl95-2) established in tissue culture. *Vitr.* **1983**, *19*, 147–158.

52. Ma, A.Y.; Xie, S.W.; Zhou, J.Y.; Zhu, Y. Nomegestrol acetate suppresses human endometrial cancer rl95-2 cells proliferation in vitro and in vivo possibly related to upregulating expression of sufu and wnt7a. *Int J Mol. Sci.* **2017**, *18*.

53. Rubin, S.C.; Federici, M.G.; Lloyd, K.O.; Lewis, J.L., Jr.; Hoskins, W.J. Characterization of a human endometrial carcinoma cell line producing intraperitoneal tumor growth in immunodeficient mice. *Gynecol. Oncol.* **1992**, *45*, 273–278.

54. Kanishi, Y.; Kobayashi, Y.; Noda, S.; Ishizuka, B.; Saito, K. Differential growth inhibitory effect of melatonin on two endometrial cancer cell lines. *J. Pineal. Res.* **2000**, *28*, 227–233.

55. Ueda, M.; Fujii, H.; Yoshizawa, K.; Abe, F.; Ueki, M. Effects of sex steroids and growth factors on migration and invasion of endometrial adenocarcinoma sng-m cells in vitro. *JPN J. Cancer Res.* **1996**, *87*, 524–533.

56. Yuan, Y.; Kim, W.H.; Han, H.S.; Lee, J.H.; Park, H.S.; Chung, J.K.; Kang, S.B.; Park, J.G. Establishment and characterization of cell lines derived from uterine malignant mixed mullerian tumor. *Gynecol. Oncol.* **1997**, *66*, 464–474.

57. Hirai, Y.; Kawaguchi, T.; Hasumi, K.; Kitagawa, T.; Noda, T. Establishment and characterization of human cell lines from a serous papillary adenocarcinoma of the endometrium. *Gynecol Oncol* **1994**, *54*, 184–195.

58. Boyd, J.A.; Rinehart, C.A., Jr.; Walton, L.A.; Siegal, G.P.; Kaufman, D.G. Ultrastructural characterization of two new human endometrial carcinoma cell lines and normal human endometrial epithelial cells cultured on extracellular matrix. *Vitr. Cell Dev. Biol.* **1990**, *26*, 701–708.

59. Ramondetta, L.; Mills, G.B.; Burke, T.W.; Wolf, J.K. Adenovirus-mediated expression of p53 or p21 in a papillary serous endometrial carcinoma cell line (spec-2) results in both growth inhibition and apoptotic cell death: Potential application of gene therapy to endometrial cancer. *Clin. Cancer Res.* **2000**, *6*, 278–284.

60. Fushiki, H.; Hidaka, T.; Fujimura, M.; Yasoshima, K.; Yamakawa, Y.; Izumi, R. Characterization of a newly established human tumor cell line (ten) from a patient with clear cell carcinoma of the uterine body and its sensitivity to anti-cancer agents. *Hum. Cell* **1997**, *10*, 199–208.

61. Santin, A.D.; Bellone, S.; Gokden, M.; Palmieri, M.; Dunn, D.; Agha, J.; Roman, J.J.; Hutchins, L.; Pecorelli, S.; O'Brien, T.; et al. Overexpression of her-2/neu in uterine serous papillary cancer. *Clin. Cancer Res.* **2002**, *8*, 1271–1279.

62. English, D.P.; Bellone, S.; Cocco, E.; Bortolomai, I.; Pecorelli, S.; Lopez, S.; Silasi, D.A.; Schwartz, P.E.; Rutherford, T.; Santin, A.D. Oncogenic pik3ca gene mutations and her2/neu gene amplifications determine the sensitivity of uterine serous carcinoma cell lines to gdc-0980, a selective inhibitor of class i pi3 kinase and mtor kinase (torc1/2). *Am. J. Obs. Gynecol.* **2013**, *209*, 465 e461–e469.

63. Black, J.D.; Lopez, S.; Cocco, E.; Bellone, S.; Altwerger, G.; Schwab, C.L.; English, D.P.; Bonazzoli, E.; Predolini, F.; Ferrari, F.; et al*.* Pik3ca oncogenic mutations represent a major mechanism of resistance to trastuzumab in her2/neu overexpressing uterine serous carcinomas. *Br. J. Cancer* **2015**, *113*, 1020–1026.
